# Solutions to the Dilemma of Antibiotics Use in Livestock and Poultry Farming: Regulation Policy and Alternatives

**DOI:** 10.3390/toxics13050348

**Published:** 2025-04-27

**Authors:** Shimei Zheng, Yongchao Li, Cuihong Chen, Naiyu Wang, Fengxia Yang

**Affiliations:** 1 College of Chemistry and Chemical and Environmental Engineering, Weifang University, Weifang 261061, China; 2 School of Environment and Natural Resources, Zhejiang University of Science and Technology, Hangzhou 310023, China; 3 Key Laboratory of Pollution Processes and Environmental Criteria (Ministry of Education), College of Environmental Science and Engineering, Nankai University, Tianjin 300350, China; 4 Agro-Environmental Protection Institute, Ministry of Agriculture and Rural Affairs, Tianjin 300191, China

**Keywords:** antibiotics, livestock and poultry farming, management policy, alternatives

## Abstract

While the application of antibiotics in livestock production has undeniably propelled the rapid growth of animal husbandry, the escalating crisis of antimicrobial resistance stemming from antibiotic use poses significant threats to global public health and sustainable agricultural development. To address this critical challenge, multifaceted strategies have been implemented through coordinated policy interventions and scientific innovations. This review systematically examines two pivotal dimensions: (1) evolving regulatory frameworks governing antibiotic usage and (2) emerging non-antibiotic alternatives, with a particular focus on their implementation mechanisms and technological maturation. The analysis of transnational antibiotic governance encompasses comparative policy evolution in the European Union, the United States, and China. These regulatory paradigms address critical control points including registration management policies, usage monitoring systems, and integrated surveillance programs. Concerning technological alternatives, six categories of antibiotic substitutes are critically evaluated: Chinese herbal formulations, plant-derived essential oils, antimicrobial peptides, microecological agents, acidifiers, and enzyme preparations. These solutions are functionally categorized into prophylactic agents (enhancing disease resilience) and zootechnical additives (optimizing feed efficiency). These antibiotic alternatives demonstrate certain efficacy in alleviating the challenges of antibiotic overuse, yet they still face multiple implementation barriers. Further investigations are warranted to establish standardized efficacy evaluation protocols and conduct technoeconomic feasibility assessments under commercial-scale production conditions. Ultimately, resolving the antibiotic dilemma requires synergistic collaboration between regulatory bodies, pharmaceutical innovators, and academic researchers. This work emphasizes the crucial interplay between evidence-based policymaking and technological advancement in shaping sustainable livestock production systems.

## 1. Introduction

The strategic application of antibiotic agents in the modern livestock and poultry system constitutes a transformative milestone in agricultural biotechnology. Within intensive livestock production systems, veterinary antibiotics serve as critical biocontrol tools, paralleling the essential functions of agrochemicals (e.g., herbicides, inorganic fertilizers) in crop cultivation. Four principal applications of antibiotics were delineated by McEwen and Fedorka-Clay (2002) [1], including therapeutic intervention, disease control measure, disease prevention, and growth promoter. The therapeutic and prophylactic applications of antibiotics in livestock and poultry disease management remain clinically essential. However, despite recommendations and regulations to the contrary, antibiotics are routinely employed in practice for both growth promotion and disease prevention in some parts of the world. Nontherapeutic antibiotics use persists in livestock operations, primarily driven by profit maximization objectives. Given the absence of professional knowledge, livestock and poultry producers face significant challenges in determining antimicrobial therapy necessity for clinically ambiguous cases.

The nontherapeutic practices induce drug residues and antimicrobial resistance (AMR), directly compromising sustainable livestock production systems [2,3]. Tetracycline resistance has been discovered in broiler chickens fed with growth-promoting antibiotics [4,5]. The transmission of AMR occurs through both direct (foodborne or contact) and indirect (environmental) pathways, posing significant public health risks. The 1969 Swann Report to the British Parliament first raised concerns about AMR in human medicine, linking animal antibiotic use to human health risks through its seminal recommendation to prohibit subtherapeutic applications in agricultural practices [6]. In the 1980s, pathogenic bacteria resistant to various antibiotics emerged globally. Groundbreaking 2001 research demonstrated zoonotic transfer of AMR determinants from livestock to human gut microbiota [7], and subsequent 2003 research confirmed AMR dissemination through foodborne transmission pathways [8]. Therefore, mounting scientific consensus advocates phasing out agricultural antibiotic prophylaxis under the One Health framework. While acknowledging historical productivity gains in intensive farming, chronic subtherapeutic administration induces multifaceted risks including AMR propagation, ecosystem contamination, residual persistence in animal-derived products, and zoonotic transmission risks [9,10,11,12,13].

The emergence of antibiotic resistance has diminished the effectiveness of antibiotic growth promoters, with studies conducted after 2000 revealing a significant decline in their efficacy compared to that before 1985 [14]. The WHO considers antibiotic resistance as one of the major threats to global public health [15]. Infection with antibiotic-resistant bacteria can prolong treatment duration, increase medical costs, and even elevate mortality risks [16,17]. It is estimated that antibiotic resistance caused 1.27 million deaths worldwide in 2019, surpassing the number of deaths attributed to AIDS and malaria [18]. If no action is taken to curb the development of resistance, it is projected that 10 million people worldwide will succumb to drug-resistant bacterial infections by 2050 [17]. Diseases frequently plunge individuals into poverty cycles, with the WHO projecting that AMR could thrust an additional 24 million people into extreme poverty by 2030 [18].

Global antibiotics use in food animal production has transcended national boundaries to become a quintessential One Health concern. Many antibiotics that belong to the last line of defense for humans have been used in agriculture and animal husbandry; necessarily, action should be taken to restrict the use of critical antibiotics. To tackle the issues of antibiotic misuse and resistance, numerous countries and organizations have implemented management systems to oversee and regulate antibiotics usage. Additionally, emerging research in veterinary pharmacology underscores the necessity of transitioning to non-antibiotic growth promoters as sustainable alternatives to circumvent AMR development in livestock systems. This work systematically evaluates multinational governance frameworks on antibiotic usage, focusing on the European Union (EU), the United States (U.S.), and China. It further critically assesses biotechnological alternatives, such as Chinese herbal formulations, plant-derived essential oils, antimicrobial peptides, microecological agents, acidifiers, and enzyme preparations. This analysis elucidates global strategies addressing antibiotic use challenges and delineates viable transition pathways toward antibiotic stewardship optimization.

## 2. Regulation Policy from Different Countries and Organizations

### 2.1. Regulation Policy from the European Union and Its Member States

**Figure 1:**
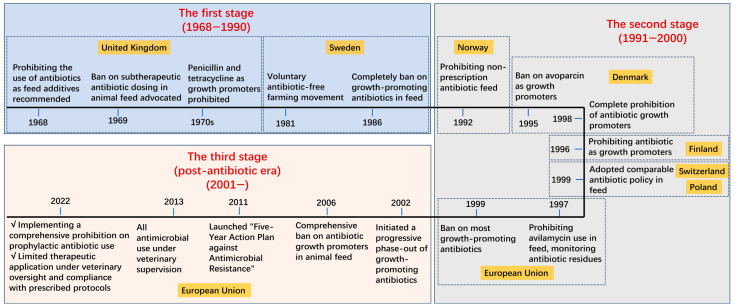
Three primary stages of regulation policy from the European Union and its member states.

In restricting the use of veterinary antibiotics, European countries have emerged as trendsetters, consistently leading global efforts through stringent regulatory policies. The management of antibiotics for growth promotion or disease prevention in Europe has evolved through three primary stages (Figure 1).

The initial phase of restricting therapeutic antibiotic additives was spearheaded by the United Kingdom. Notably, Britain became the first nation to propose and implement a formal “no antibiotics” policy. In 1968, the UK’s Swann Committee recommended prohibiting the use of antibiotics with human therapeutic applications as feed additives in livestock production [6]. The subsequent Swann Report (1969), published by the British Parliament, established a scientific link between agricultural antibiotic use and the rise of antibiotic-resistant infections in humans [6]. In response, the Swann Committee advocated a ban on subtherapeutic antibiotic dosing in animal feed. By the early 1970s, the UK had prohibited penicillin and tetracycline as growth promoters [19,20,21]. This regulatory momentum extended to Scandinavia when, in 1981, the Swedish Farmers’ Association initiated a voluntary antibiotic-free farming movement. Their advocacy culminated in 1986 with Sweden becoming the first nation to legislate a complete ban on growth-promoting antibiotics in feed—a policy driven by mounting public concerns over drug residues and AMR [22,23,24].

The second stage began in the 1990s, marked by further restrictions on approved antibiotic use. Norway took pioneering action in 1992 by prohibiting non-prescription antibiotic feed additives, achieving a dramatic reduction in agricultural antibiotic consumption—from 50 metric tons in 1987 to less than 5 metric tons by 1993 [25]. Denmark demonstrated a stark disparity in antibiotic usage in 1993. Only 22 kg of avoparcin—a reserve-class antibiotic designated for critical clinical scenarios (e.g., life-threatening multidrug-resistant bacterial infections)—was allocated for therapeutic use, whereas 19,472 kg was utilized as a growth promoter in livestock and poultry farming systems [26]. Denmark implemented progressive restrictions on antibiotic growth promoters beginning with the 1995 ban on avoparcin. The government simultaneously introduced fiscal disincentives through voluntary taxation measures targeting feed manufacturers. This policy framework culminated in the complete prohibition of antibiotic growth promoters by 1998. While therapeutic antibiotic use experienced a temporary increase following the 1995 restrictions, aggregate consumption of growth-promoting antibiotics demonstrated a dramatic decline—falling from 115,786 kg in 1994 to just 12,283 kg by 1999 [27,28]. Following Finland’s 1996 precedent, both Poland and Switzerland adopted comparable regulatory measures in 1999 [29]. The EU intensified its regulatory framework in 1997 by prohibiting avilamycin use in livestock feed across all member states, while simultaneously instituting mandatory surveillance through regular sampling of animal products to monitor antibiotic residues and resistance patterns [30]. This regulatory trajectory culminated in 1999 with the EU’s comprehensive ban on most growth-promoting antibiotics.

The 21st century marked the third phase of antibiotic restrictions, ushering in what is now termed the post-antibiotic era. This period has been characterized by expanded prohibitions on antibiotic use and increasingly stringent regulatory measures. In 2002, the European Council initiated a progressive phase-out of all growth-promoting antibiotics, culminating in the EU’s comprehensive 2006 ban on antibiotic growth promoters in animal feed. Concurrently with these restrictions, technological advancements enabled the EU to implement regulated protocols for prophylactic antibiotic use in livestock. Demonstrating continued commitment to antibiotic stewardship, the Netherlands established ambitious reduction targets in 2009, mandating a 50% decrease in veterinary antibiotic usage between 2009 and 2013 [31]. In 2011, the European Commission launched its “Five-Year Action Plan against Antimicrobial Resistance” to strengthen regulatory oversight of antibiotic use in both human medicine and animal husbandry. This initiative specifically targeted improved governance of veterinary antibiotics. Building upon this framework, the European Medicines Agency (EMA) implemented stringent prescription requirements in 2013, mandating that all antimicrobial use in livestock and poultry operations for disease prevention be conducted under veterinary supervision. EMA surveillance data from 2005 to 2009 revealed significant progress in antibiotic reduction across nine European nations (Czech Republic, Denmark, Finland, France, the Netherlands, Norway, Switzerland, Sweden, and the United Kingdom). Total antibiotic consumption in these countries declined by 11%, from 2513 metric tons in 2005 to 2232 metric tons in 2009 [32] Among the 29 European countries reporting antibiotic usage data between 2010 and 2014, only seven demonstrated increased consumption, while the remaining nations achieved significant reductions [33]. The EU further strengthened its regulatory framework by implementing a comprehensive prohibition on prophylactic antibiotic use in January 2022 [34]. Under exceptional circumstances where infection risk is substantiated, limited therapeutic application remains permitted; however, such use requires strict veterinary oversight and compliance with prescribed protocols.

The Danish regulatory approach to antibiotic use in livestock production offers valuable lessons. Denmark’s progressive restrictions began with the livestock industry’s voluntary discontinuation of all antibiotic growth promoters in 1998, followed by the mandatory prohibition of these substances in swine production the subsequent year. To reinforce these measures, the Danish government implemented stringent controls on antibiotic sales and enhanced veterinary prescription requirements [35]. Since 2010, the Danish government has implemented a progressive “yellow card” program for pig and cattle farming with the objective of reducing veterinary antibiotic use by 10%. This regulatory framework mandates that (1) all antibiotic administration must be veterinarian-prescribed; (2) veterinarians are prohibited from profiting directly from antibiotic sales; and (3) pharmacies, veterinarians, and producers must systematically report antibiotic sales and usage data. Producers who exceed twice the industry’s average antibiotic consumption from the previous year receive a “yellow card” warning, subjecting them to financial penalties and enhanced regulatory scrutiny [36]. Non-compliant operations may face mandatory reductions in herd size [35]. Complementing these measures, Denmark’s Ministry of Food and Agriculture established the Danish Integrated Antimicrobial Resistance Monitoring and Research Programme (DANMAP) prior to implementing its comprehensive ban. This surveillance system systematically tracks and analyzes antibiotic utilization patterns across livestock, poultry, and human populations, providing critical data for evidence-based policy formulation. While the EU’s prohibition of antimicrobial growth promoters in animal feed is fundamentally driven by evidence linking veterinary antimicrobial use to zoonotic AMR, the net impact of this policy intervention on agricultural productivity and anthroponotic health risks continues to generate scientific and socioeconomic discourse.

The prohibition of antibiotic growth promoters in animal feed has yielded significant unintended consequences for livestock health and disease management. The EU’s prohibition on nontherapeutic antibiotics use in animal production has, particularly during the initial implementation year, resulted in elevated therapeutic antimicrobial consumption, a reduction in zootechnical performance, and a heightened prevalence of infectious diseases in food animals. Notably, swine production has experienced elevated mortality rates among early-weaned piglets due to diarrheal pathogens such as *Escherichia coli* and *Lawsonia intracellularis*, while poultry operations have observed increased incidence of necrotizing enteritis caused by *Clostridium perfringens*. These emerging health challenges have consequently driven substantial increases in therapeutic antimicrobial usage [30]. This regulatory shift has profoundly impacted the animal health industry. Pharmaceutical innovation in veterinary medicine has been constrained by three key factors: (1) heightened government regulatory oversight, (2) more stringent approval criteria, and (3) elevated safety and efficacy standards for new drug evaluations. Furthermore, epidemiological surveillance data suggest that the EU’s antibiotic restrictions may have contributed to increased human infection rates of *Salmonella* and *Campylobacter jejuni*, with these pathogens demonstrating enhanced resistance profiles to tetracyclines, sulfonamides, and fluoroquinolones [30].

Long-term epidemiological monitoring demonstrates that the EU’s prohibition of antibiotic growth promoters has achieved its primary objective of reducing overall antimicrobial consumption while maintaining livestock production efficiency. Following implementation in Denmark, Norway, and Sweden, aggregate antibiotic use in food animals declined significantly without compromising productivity metrics. A comprehensive Danish evaluation (1992–2008) revealed sustained improvements in swine production parameters post-ban, including enhanced yield, increased average daily weight gain in weaned and fattening piglets, and stable mortality rates [37]. Sweden’s surveillance data show particularly striking outcomes, with a 55% reduction in veterinary antibiotic utilization accompanied by persistently low AMR indices in food animal populations [38]. 

### 2.2. Regulation Policy from the United States

The U.S. employs a distinct regulatory framework for growth-promoting and prophylactic antibiotics in animal agriculture, characterized by multi-agency oversight. The U.S. FDA, the U.S. Department of Agriculture (U.S. DA), and the U.S. Centers for Disease Control and Prevention (CDC) collectively administer a comprehensive surveillance system for veterinary antimicrobial use. This interagency approach provides stratified safeguards through diversified regulatory mechanisms designed to (1) monitor antibiotic residues, (2) mitigate resistance development, and (3) protect public health.

The U.S. implemented antibiotic use regulations later than European nations, largely due to industry resistance. In 1977, the U.S. FDA initially proposed prohibiting penicillin and tetracycline as feed additives for therapeutic purposes. However, Congress deferred immediate action pending further research, citing limitations in contemporary analytical methodologies. The U.S. approach to antibiotic reduction in livestock production has evolved through two distinct phases: The initial regulatory period (1995–2006) mirrored European policies by systematically eliminating certain subtherapeutic antibiotic applications in animal feed [39]. Subsequently (2006–present), the market became bifurcated into conventional and antibiotic-free production systems, with substantial institutional support emerging for antibiotic-free operations [40].

The initial regulatory phase (1995–2006) focused on implementing targeted restrictions on antibiotic use in animal production. While adopting key elements of the EU’s antibiotic reduction framework, the U.S. FDA adapted these policies to domestic contexts through selective implementation. For example, when the EU prohibited zinc bacteriocins in feed in 1999 due to their growth-promoting effects, the U.S. maintained their approved use as feed additives while concurrently joining the EU in banning virginiamycin. During this period, the U.S. systematically reduced subtherapeutic antibiotic applications in livestock feed, culminating in the complete elimination of growth-promoting tylosin and monensin by 2006.

The second regulatory phase (2006–present) represents a transition from antibiotic restrictions to comprehensive prohibitions, coupled with active promotion of antibiotic-free production systems. Since 2006, the U.S. Congress has enacted legislation aligning with European models to strengthen veterinary antibiotic governance [41]. In 2013, the U.S. FDA introduced its Antibiotic Stewardship Initiative, establishing two fundamental principles: (1) antimicrobials should only be administered when clinically necessary for treating diagnosed conditions, and (2) all antibiotic use must occur under veterinary supervision [42]. This policy shift specifically targeted the previously unregulated over-the-counter availability of medically important antibiotics, mandating veterinary prescriptions for their procurement [42]. The U.S. FDA’s regulatory framework adopted a gradual approach; while permitting medicated feeds during the transition period, it actively incentivized antibiotic-free production, defined as complete avoidance of both growth-promoting and subtherapeutic antibiotic applications [43]. Several milestones followed thereafter. In 2014, subtherapeutic antibiotic use in livestock feed was prohibited. In 2017, all antibiotic feed supplements were banned, and veterinary oversight for water/feed medication became mandatory [42,44,45]. In 2023, veterinary prescriptions for all antibiotic administration routes became a requirement (including injectables) [46].

Concerning therapeutic antibiotic use, regulatory agencies have implemented targeted reduction strategies based on the potential risks of antibiotic residues to human health. The implemented measures include the following key policy interventions. In 2018, the U.S. DA introduced a focused program to decrease the usage of high-priority antibiotics like norfloxacin [47]. In 2019, building on prior efforts, regulators expanded reduction targets to include critical veterinary cephalosporins such as cefotaxime sodium [48]. In 2020, a specialized framework was established for antibiotics with dual human–animal applications, particularly targeting essential medications like erythromycin to preserve clinical efficacy [49].

The U.S. FDA enforces a rigorous multi-stage approval protocol for antimicrobials used in animal production. The regulatory process has the following requirements: (1) Scientific Evaluation Phase. Candidate antimicrobials must demonstrate safety and efficacy through standardized testing. The Center for Veterinary Medicine (CVM) conducts a comprehensive review of all experimental data. Compounds must meet established safety margins and therapeutic performance benchmarks. (2) Regulatory Submission Process. Successful applicants submit formal documentation that includes complete compound characterization data, proposed labeling specifications, and manufacturing quality control protocols. CVM performs independent verification of all claims. (3) Approval Criteria. Final authorization requires conclusive evidence of target animal safety, clinical effectiveness, and human food safety assurance [50]. A critical component of the approval framework is the establishment of mandatory withdrawal periods, the minimum interval between final antibiotic administration and slaughter. These scientifically determined intervals ensure tissue residue levels remain below established tolerance thresholds, thereby guaranteeing food safety compliance. The U.S. FDA maintains continuous post-market surveillance to verify ongoing adherence to these safety parameters [50].

The U.S. maintains a comprehensive surveillance system for monitoring both antibiotic residues and AMR in meat products. This dual-track approach involves the following: (1) A residue monitoring program, which is conducted by the U.S. DA’s Food Safety and Inspection Service (FSIS) at slaughterhouses and ports of entry. Systematic sampling and analysis of meat products are performed to test for antibiotic residues and other prohibited substances. Public reporting of violations through the FSIS Residue Violation Information System (RVIS) ensures regulatory transparency. (2) AMR surveillance, which is implemented through the National Antimicrobial Resistance Monitoring System (NARMS) established in 1996. The surveillance system focuses on enteric bacteria from food animals to track resistance patterns.

The U.S. DA has established a mandatory labeling system for veterinary antibiotics to address the economic challenges of antibiotic-free production. This regulatory framework has the following requirements: (1) Clear product differentiation. All medically important therapeutic antibiotics in animal production are explicitly labeled. Mandatory veterinary prescription is required for labeled antibiotics. (2) Market-based incentive structure. Formal recognition of price premiums certifies antibiotic-free products, and transparent pricing mechanisms reflect production cost differentials between conventional livestock operations utilizing antibiotics and certified antibiotic-free production systems. (3) Dual policy objectives. They not only facilitate informed purchasing decisions through standardized labeling but also ensure financial viability for producers transitioning to antibiotic-free practices through market compensation. This approach leverages market forces to achieve public health goals while maintaining agricultural economic stability. The labeling system serves as both a regulatory compliance mechanism and a market signal, aligning consumer behavior with antimicrobial stewardship objectives.

### 2.3. Regulation Policy from China

China’s regulatory experience with antibiotic prohibition remains comparatively recent within the global context. The nation’s agricultural modernization since the 1980s, characterized by the adoption of large-scale intensive farming systems, precipitated a substantial rise in veterinary antibiotic utilization. However, the consequent AMR crisis only emerged as a significant public health concern during the 1990s. A pivotal regulatory milestone occurred in 2015 when the Ministry of Agriculture (now the Ministry of Agriculture and Rural Affairs, MARA) implemented restrictions on four classes of fluoroquinolone veterinary drugs (including lomefloxacin, pefloxacin, norfloxacin, and ofloxacin) in food animal production. This policy intervention marked China’s initial substantive step toward establishing comprehensive antibiotic use regulations.

On 9 July 2019, the MARA promulgated Announcement No. 194, mandating the market withdrawal of all growth-promoting medicinal feed additives (with the exception of traditional Chinese medicine formulations) effective 1 January 2020 [51]. All veterinary pharmaceutical products manufactured or imported prior to the regulatory cutoff date shall retain market authorization for commercial distribution through 31 December 2020. Effective 1 July 2020, all Chinese feed manufacturers were required to discontinue production of growth-promoting antibiotic additives, with traditional Chinese medicine preparations remaining exempt. This regulatory milestone signified China’s formal transition to a comprehensive antibiotic-free feed policy framework. Concurrently, regulatory authorities have implemented two complementary measures: (1) a comprehensive revision of product classification codes for antibiotic feed additives and (2) the development of standardized quality specifications for traditional Chinese medicine-based alternatives. China’s antibiotic restriction policy has undergone progressive intensification, manifesting in two key dimensions: (1) continuous expansion of regulatory scope and enforcement intensity and (2) successively stringent compliance requirements imposed on feed processing enterprises [52].

These policy measures signify that since 2020, pharmaceutical feed additives have been completely phased out from animal feed formulations, with their application now strictly limited to therapeutic purposes in livestock and poultry production under veterinary supervision. The National Action Plan for Combating Antimicrobial Resistance (2022–2025) stipulates that all non-prohibited antibiotics must be used under veterinary supervision and establishes a target of achieving prescription veterinary antibiotic sales exceeding 80% by 2025. In China, the consumption of veterinary antibiotics reversed its previous upward trend in 2014 and has since demonstrated a consistent year-on-year decline. Antibiotic usage decreased by 53%, from 69,292 metric tons in 2014 to approximately 32,500 metric tons in 2021 [53].

### 2.4. Other Countries and Organizations

Following the EU’s prohibition on nontherapeutic antibiotic use in agriculture, multiple nations have enacted analogous policies to phase out growth-promoting antibiotics. In 2008, Japan implemented a complete ban on the use of all antibiotics as feed additives. The country also adopted the “National Action Plan on Antimicrobial Resistance (2016–2020)”, which established an integrated One Health surveillance system for monitoring antimicrobial-resistant bacteria isolated from humans, animals, food, and the environment [54]. In July 2011, the South Korean Ministry of Agriculture, Forestry, Livestock and Food enforced a strict antibiotic ban, prohibiting the use of all antibiotic growth promoters [55]. Australia has prohibited the use of fluoroquinolones in food-producing animals [56]. International cooperation on AMR persists, with active collaborations among key stakeholders such as Japan, the U.S., and the EU [57].

## 3. Antibiotic Alternatives

**Figure 2:**
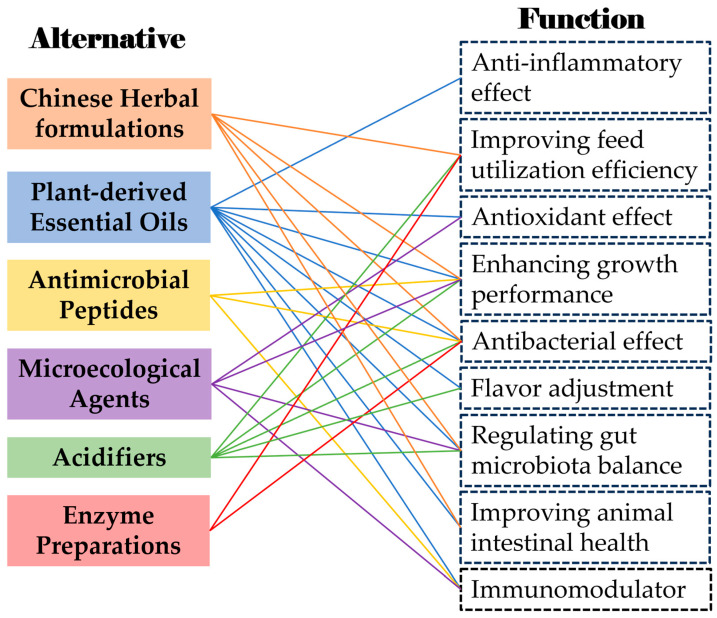
The alternatives to antibiotics and their functions.

Alternative antibiotic products include Chinese herbal preparations, plant essential oils, antimicrobial peptides, microecological agents, acidifying agents, and enzyme preparations [58,59]. To restrict and reduce antibiotic use in animal production, research efforts are being directed toward two parallel strategies: (1) the development of novel antibiotics and (2) the advancement of antibiotic alternatives [60]. Growing environmental and public health concerns have propelled the emergence of green, non-toxic, and residue-free feed additives as both a prominent research focus and an industry development priority in modern animal husbandry [59,61]. This represents a critical challenge requiring urgent attention within the livestock sector. This study systematically evaluates several representative antibiotic alternatives, including Chinese herbal formulations, plant-derived essential oils, antimicrobial peptides, microecological agents, acidifiers, and enzyme preparations (Figure 2).

### 3.1. Chinese Herbal Formulations

Chinese herbal medicines contain diverse bioactive compounds that serve dual roles as both therapeutic agents and natural feed additives. These bioactive compounds demonstrate significant potential for the prevention and treatment of bovine mastitis. Their antimicrobial mechanisms parallel those of conventional antibiotics, while offering the distinct advantage of eliminating antibiotic residue concerns in milk products [62]. The Ministry of Agriculture and Rural Affairs of China (MARA) officially recognized 117 natural plant species as approved feed ingredients in its Announcement No. 1773 “Catalogue of Feed Raw Materials”, sanctioning their use in animal nutrition and health products. These herbal formulations offer multiple advantages: (1) demonstrated safety and reliability, (2) absence of toxic side effects, and (3) no risk of inducing microbial resistance, positioning them as leading alternatives to antibiotic growth promoters. MARA has systematically approved specific veterinary traditional Chinese medicine formulations for long-term use through official announcements, with detailed specifications provided in Table 1.

Botanical supplements with gastroprotective and digestive properties, including cinnamon (*Cinnamomum verum*), basil (*Ocimum basilicum*), amaranth (*Amaranthus* spp.), coriander (*Coriandrum sativum*), and rosemary (*Rosmarinus officinalis*), can serve as effective growth promoters in livestock production [63,64]. When administered in appropriate doses, these phytogenic additives significantly improve feed utilization efficiency. Studies demonstrate that bioactive compounds isolated from traditional Chinese medicine (e.g., organic acids, tannins, and polysaccharides) enhance broiler performance metrics, particularly by improving feed conversion ratios and reducing feed-to-gain ratios [65]. Furthermore, fermented herbal formulations containing *Schisandra chinensis*, *Plantago asiatica*, and ginseng (*Panax ginseng*) stems/leaves, when inoculated with lactic acid bacteria and fed to 35-day-old weaned piglets, yield multiple benefits including increased apparent nutrient digestibility, enriched populations of beneficial gut microbiota, improved intestinal health status, and enhanced overall growth performance [66].

Certain herbal medicines with detoxifying properties, including *Lentinula edodes*, *Tremella fuciformis*, *Astragalus membranaceus*, and *Artemisia annua*, exhibit potent antibacterial effects. These botanicals can effectively suppress pathogenic bacteria in livestock and poultry intestines while promoting intestinal microbiota homeostasis [67]. Chinese herbal medicine additives such as *Rheum officinale*, *Phellodendron amurense*, and *Coptis chinensis* demonstrate inhibitory activity against *Staphylococcus aureus* [68]. Furthermore, phytogenic feed additives containing *Andrographis paniculata*, *Camellia sinensis*, and *Portulaca oleracea* exhibit therapeutic efficacy against epidemic diarrhea virus infections [69].

The animal medicine industry faces several critical challenges regarding traditional Chinese herbal medicines [60]: (1) inconsistent therapeutic efficacy and quality due to seasonal variations, geographical differences in production regions, and diverse processing methods; (2) the current practice of direct crushing and mixing as feed additives, which results in high inclusion rates and elevated feed costs; and (3) inadequate quality control standards for veterinary applications, creating difficulties in regulating dosage precision and therapeutic outcomes. To address these issues, a comprehensive scientific evaluation system must be developed to optimize herbal formulation combinations for livestock disease prevention/treatment, establish precise administration protocols, and improve cost-effectiveness while maintaining therapeutic efficacy.

### 3.2. Plant-Derived Essential Oils

Plant-derived essential oils are volatile aromatic liquids typically extracted from various botanical tissues including buds, flowers, leaves, stems, fruits, seeds, and bark. Chemically, they primarily consist of lipophilic secondary metabolites. The bioactive components can be classified into two major categories: (1) phenylpropanoid derivatives (e.g., eugenol, cinnamaldehyde), with over 50 identified variants, and (2) terpenoid derivatives (e.g., thymol, carvacrol, aliphatic compounds), comprising more than 4000 known structures [59,61,70].

Plant essential oils have functions such as antibacterial, anti-inflammatory, and antioxidant effects, immune regulation, increasing feed flavor, regulating gut microbiota balance, improving animal intestinal health, and enhancing animal growth performance [70,71]. Specific formulations demonstrate targeted benefits. For example, composite essential oils containing allicin, beef tallow, cinnamon oil, and thyme oil significantly enhance antioxidant capacity in weaned piglets, an effect attributable to their high phenolic content [72]. And *Forsythia suspensa* oil effectively mitigates corticosterone-induced oxidative stress in broilers by reducing malondialdehyde (MDA) levels and enhancing total antioxidant capacity [73].

Plant essential oils can stimulate lymphocytes in the intestinal mucosal lamina propria, enhance humoral and cellular immunity, and improve the immunity and intestinal mucosal barrier function of livestock and poultry [70]. Eucalyptus essential oil, rich in 1,8-cineole (eucalyptol), demonstrates significant immunostimulatory capacity when supplemented in feed, increasing macrophage phagocytic activity, elevating peripheral blood monocyte populations, and upregulating CD44 receptor expression on immune cells. Terpinen-4-ol, the primary bioactive component of tea tree oil, exhibits dual protective effects. The first effect involves enhancing intestinal epithelial integrity by upregulating tight junction protein expression, thereby ameliorating dextran sulfate sodium (DSS)-induced colitis in murine models. The second effect consists in mitigating heat stress-induced intestinal damage and suppressing the overexpression of inflammatory cytokines following oral administration [74].

The active ingredients of plant essential oils, such as phenols, alcohols, aldehydes, ketones, and ethers, have good antibacterial activity [70]. Their efficacy primarily stems from lipophilicity, which enables penetration of bacterial cell walls and plasma membranes, leading to membrane disruption and structural damage to intracellular components. Cinnamon-derived compounds, for instance, demonstrate antibacterial action through multiple mechanisms: (1) alteration of protein conformation, (2) degradation of cell wall integrity, (3) induction of cytoplasmic leakage, (4) increased membrane permeability, and (5) cellular deformation [75].

However, the practical application of plant essential oils is constrained by their chemical instability, including sensitivity to environmental factors and high volatility, necessitating stringent temperature control during storage and handling [70] Furthermore, the strong aromatic profiles of certain essential oils may negatively impact feed palatability and animal intake. To mitigate these limitations, encapsulation techniques (e.g., microencapsulation), molecular separation, or adsorbent-based stabilization methods are often employed to mask undesirable odors while preserving bioactive properties.

### 3.3. Antimicrobial Peptides

Antimicrobial peptides represent a class of small-molecular-weight proteins derived from diverse biological sources including microorganisms, fungi, plants, and animals. These peptides exhibit several advantageous characteristics: (1) structural diversity, (2) broad-spectrum bioactivity (encompassing antibacterial and anti-inflammatory properties), (3) growth-promoting effects, (4) remarkable stability under high temperatures and acidic conditions, and (5) excellent aqueous solubility [60,76,77,78].

Antimicrobial peptides serve as critical immunomodulators in animals, actively promoting the differentiation and maturation of dendritic cells and macrophages while enhancing T lymphocyte functionality [79]. Beyond their immunological roles, antimicrobial peptides significantly improve growth performance and production metrics in livestock. Poultry studies demonstrate that dietary antimicrobial peptides supplementation markedly increases average daily gain while reducing feed conversion ratios [80,81]. Similarly, in weaned piglets, antimicrobial peptides have been shown to accelerate growth rates, improve feed efficiency, and enhance immune competence [82].

Antimicrobial peptides exhibit bactericidal activity through both membrane-disruptive and non-membrane-mediated mechanisms [76,77]. Their cationic properties confer selective affinity for anionic components of microbial membranes, including lipopolysaccharides in Gram-negative bacteria, teichoic acids in Gram-positive bacteria, and mannans in fungal cell walls. This interaction induces membrane permeabilization or pore formation, leading to either leakage of cellular components or intracellular penetration to exert cytotoxic effects [83]. A notable example is Polybia-MP1, a 14-residue antimicrobial peptide derived from wasp venom, which demonstrates potent activity against multidrug-resistant pathogens (including *Staphylococcus aureus*, *Escherichia coli*, and *Klebsiella pneumoniae*) isolated from bovine mastitis cases [84].

Despite their therapeutic potential, antimicrobial peptides face several limitations: (1) technically challenging extraction processes with low yields and high production costs; (2) insufficient understanding of their mechanisms of action; and (3) potential safety risks including hypersensitivity reactions and hemolysis in livestock [60,76,77,78]. These constraints necessitate comprehensive investigations into optimized extraction/isolation methodologies, synthetic production techniques, pharmacokinetic properties, mechanistic pathways, and in vivo toxicity profiles to facilitate clinical translation. Additionally, emerging evidence indicates that bacteria can develop resistance to antimicrobial peptides, with concerning reports of cross-resistance to conventional antibiotics [85].

### 3.4. Microecological Agents

Microecological preparations primarily consist of three categories: probiotics, prebiotics, and synbiotics. Based on their biological composition, these preparations can be further classified into several types, including Lactobacillus strains, Bacillus species, yeast derivatives, oligosaccharides, and microalgae-based products. These agents demonstrate high safety profiles, exhibiting no pathogenic potential and minimal adverse effects while maintaining comprehensive biological functionality. These advantageous characteristics position microecological preparations as a highly promising antibiotic alternative in animal production systems.

Microecological agents play a significant role in maintaining the balance of gut microbiota, promoting immune system development, and improving antioxidant performance. They can effectively improve the production performance and health status of animals and are conducive to the high-quality products [60]. The co-fermentation product of *Lactobacillus* and *Clostridium butyricum* demonstrates effective inhibition against intestinal pathogens and significantly reduces diarrhea incidence in weaned piglets [86]. The fermentation metabolites of *Bacillus subtilis* and *Bacillus licheniformis* enhance growth performance in broiler chickens while mitigating Clostridium perfringens-induced necrotic enteritis [87]. In dairy cows, probiotics showed the potential to treat mastitis which was induced by intestinal flora [88]. *Bacillus subtilis*, fed to heifers and transitional cows for 3 weeks before calving and throughout lactation, reduced the incidence of clinical mastitis and days of discarded milk [89].

Microecological agents serve as important immunomodulators in animal production systems, stimulating the production of immune cells, activating macrophages, and promoting the immune response in livestock and poultry. Experimental evidence demonstrates that dietary supplementation with *Lactobacillus delbrueckii* promotes dendritic cell maturation and activation, thereby improving intestinal immunoregulatory capacity in weaned piglets. Furthermore, the colonization density and population dynamics of *Lactobacillus* spp. in the gastrointestinal tract have been shown to critically affect the development and function of intestinal immune cells [90,91].

Currently, the microecological preparation industry faces several key challenges, including inconsistent product quality, insufficient viable bacterial counts, and excessive moisture content, all of which significantly constrain the efficacy of these preparations [60]. To address these limitations, comprehensive optimization of production protocols, storage conditions, transportation methods, and administration approaches is urgently required.

### 3.5. Acidifiers

Acidifiers can be classified into two main categories, single acidifiers and compound acidifiers, respectively. Single acidifiers comprise both inorganic acids (e.g., phosphoric acid, hydro-chloric acid) and organic acids (e.g., formic acid, lactic acid, fumaric acid). Compound acidifiers include phosphoric acid-based formulations, lactic acid-based blends, and organic acid salt combinations. When incorporated into animal feed, these acidifiers effectively lower the gastrointestinal pH, exerting antimicrobial effects against pathogenic microorganisms while enhancing feed digestibility and nutrient utilization. Consequently, acidifiers have gained widespread application across various livestock sectors, including poultry, swine, beef cattle, dairy cows, and sheep production [60,92].

When administered in appropriate doses, acidifiers demonstrate multiple beneficial effects on animal nutrition and health. These compounds enhance feed palatability, thereby increasing voluntary feed intake and improving growth performance. By reducing gastric pH, acidifiers create a more stable acidic environment that optimizes digestive enzyme activity and enhances nutrient digestibility [60]. This physiological modification promotes the proliferation of beneficial gut microbiota while suppressing pathogenic microorganisms such as *Escherichia coli*, ultimately reducing piglet diarrhea incidence and mitigating weaning stress [93]. The nutritional benefits are further evidenced by improved weight gain, feed efficiency, and nutrient absorption—outcomes strongly correlated with the establishment of favorable gut microflora [94]. Certain organic acids (e.g., citric acid, fumaric acid) actively participate in metabolic pathways, notably the tricarboxylic acid cycle, while lactic acid serves as both a glycolytic end-product and gluconeogenic substrate for energy production [92]. Thus, beyond their antimicrobial properties, acidifiers directly contribute to nutrient metabolism and utilization in livestock.

However, the efficacy of acidifiers demonstrates significant variability, being highly susceptible to influencing factors including animal age and husbandry conditions, as well as acidifier type and dosage. Furthermore, their large-scale application remains constrained by high production costs. Consequently, future research should prioritize the development of tailored acidification strategies, with customized formulations designed for specific livestock species and growth stages, incorporating differentiated modes of action.

### 3.6. Enzyme Preparations

Enzyme preparations are biologically active proteins that demonstrate highly efficient catalytic capabilities following specialized processing. These biocatalysts, primarily derived from plant and animal sources, can be categorized as single-enzyme preparations (e.g., cellulase, phytase) or complex enzyme formulations (e.g., stearin). Characterized by their substrate specificity and catalytic efficiency, enzymatic preparations have gained widespread application across multiple industries [60]. Particularly in animal nutrition, the feed sector has become the fastest-growing and most commercially significant market for these biocatalysts. Conservative estimates indicate that global enzyme utilization in animal feed could generate annual savings exceeding USD 8 billion in nutritional input costs [60].

Dietary enzyme supplementation enhances livestock performance by improving the digestion of otherwise indigestible feed components, thereby increasing both feed utilization efficiency and nutrient absorption. For poultry, phytase supplementation has been shown to significantly improve mineral bioavailability in laying hens, supporting sustained egg production [95]. In swine nutrition, phytase addition enhances phosphorus digestibility from plant-based ingredients while reducing both phosphorus excretion and feed costs [96]. Furthermore, the synergistic combination of amylase and protease demonstrates marked improvements in overall nutrient digestibility. This enzymatic synergy suggests potential for increased milk yield in dairy cattle through enhanced fiber and starch digestion [97].

Enzyme preparations demonstrate antimicrobial properties through substrate de-composition, effectively inhibiting intestinal pathogen growth while improving gut health and immune function in livestock. For instance, dietary supplementation with lysozyme in weaned piglets has been shown to increase fecal Lactobacillus counts and reduce both fecal scores and *Escherichia coli* populations, thereby significantly modifying the gut microbiota [98]. Similarly, xylanase supplementation exerts prebiotic effects by modulating the intestinal microbiome and enhancing immune responses. When added to corn–soybean meal diets, xylanase elevates cecal short-chain fatty acid concentrations while markedly increasing the abundance of beneficial microbiota including *Ruminococcus*, *Lactobacillus salivarius*, and *Spirochaetaceae* [99].

A key challenge in the feed enzyme industry lies in the instability of enzyme preparations under varying environmental conditions, including temperature, humidity, and pH levels during practical application, which often prevents optimal performance [60]. Future research should therefore focus on elucidating the mechanisms of action and optimizing formulation technologies to develop more stable and broadly applicable enzyme products.

## 4. Conclusions

Addressing antibiotic management requires more than just new policies and regulations; it also necessitates adopting proven frameworks and fostering cross-sectoral collaboration. The EU, the U.S., and other regions have already implemented comprehensive regulatory systems for the environmental management of veterinary drugs, supported by robust monitoring and evaluation standards. Effective antibiotic resistance management also hinges on interdisciplinary cooperation. To this end, research and academic platforms should prioritize thematic studies, incentivize interdisciplinary initiatives, and facilitate data sharing across research domains. Such collaboration enables experts from diverse fields to conduct joint research, thereby generating an evidence-based foundation for holistic antibiotic resistance management and accelerating the translation of basic research into actionable policies.

The Chinese government has proactively introduced and implemented a series of regulatory policies while coordinating cross-sectoral efforts to curb antibiotic use. However, the current antibiotic management framework still exhibits notable shortcomings. While existing policies have outlined the overarching direction for reforming antibiotic use in livestock and poultry production, they lack detailed guidance on implementation. Future policy improvements should address practical challenges in antibiotic research and development, production, application, and surveillance to further mitigate misuse in animal farming and facilitate the transition toward sustainable, low-antibiotic production systems. Given China’s relatively late focus on antibiotic regulation, resolving the issue of antibiotic overuse in livestock and poultry farming will require a long-term, phased approach rather than immediate solutions. Therefore, achieving a comprehensive ban on antibiotics remains a distant goal, necessitating sustained efforts.

Generally, antibiotic alternatives can be categorized into two groups: those for disease prevention and those for growth promotion, though these functions may overlap. Currently, probiotics, enzyme preparations, and plant extracts are among the most widely studied substitutes, demonstrating efficacy in disease prevention and treatment without known toxic side effects. Some alternatives even exhibit comparable or superior performance to antibiotics in enhancing livestock productivity, highlighting their potential for widespread adoption. However, further research is essential to facilitate the transition from antibiotics to these substitutes in practical applications.

In the post-antibiotic era, effective antibiotic stewardship and alternative therapeutic strategies represent two critical approaches to addressing the challenges of antibiotic resistance and misuse.

## Figures and Tables

**Table 1:** Veterinary drug formulations made from Chinese herbs allowed for long-term addition and use by MARA.

Veterinary Drug Formulations	Notice Number	Description of Functions
*Ligustrum lucidum* extract powder	No. 187 in 2019	Enhancing immunity and promoting growth; to promote the growth of chicken.
*Macleaya cordata* powder	No. 246 in 2019	Antibacterial, anti-inflammatory, appetizing, and growth promoting; to promote the growth of pigs, chickens, meat ducks, freshwater fish, shrimp, crabs, and turtles.
*Scutellaria baicalensis* extract powder	No. 246 in 2019	Anti-inflammatory, antibacterial, and growth promoting; to promote the growth of broiler chickens and weaned piglets.
*Callicarpa nudiflora* powder	No. 327 in 2020	Anti-inflammatory, antibacterial, hemostatic, and growth promoting; to promote the growth of pigs.
*Leonurus japonicus* extract powder	No. 408 in 2021	Promoting blood circulation and removing blood stasis; decreased egg production rate in the late stage of laying hens.
Qiweng Huangbo Powder	No. 520 in 2022	Anti-inflammatory, anti-diarrheal, and growth promoting; to prevent piglet diarrhea and improve growth performance

## Data Availability

No new data were created or analyzed in this study. Data sharing is not applicable to this article.
